# Monitoring trends in HIV prevalence among young people, aged 15 to 24 years, in Manicaland, Zimbabwe

**DOI:** 10.1186/1758-2652-14-27

**Published:** 2011-05-24

**Authors:** Kimberly A Marsh, Constance A Nyamukapa, Christl A Donnelly, Jesus M Garcia-Calleja, Phillis Mushati, Geoffrey P Garnett, Edith Mpandaguta, Nicholas C Grassly, Simon Gregson

**Affiliations:** 1 Department of Infectious Disease Epidemiology, Imperial College London, UK; 2 Biomedical Research and Training Institute, Harare, Zimbabwe; 3 MRC Centre for Outbreak Analysis and Modelling, Imperial College London, UK; 4 World Health Organization, Geneva, Switzerland

## Abstract

**Background:**

In June 2001, the United Nations General Assembly Special Session (UNGASS) set a target of reducing HIV prevalence among young women and men, aged 15 to 24 years, by 25% in the worst-affected countries by 2005, and by 25% globally by 2010. We assessed progress toward this target in Manicaland, Zimbabwe, using repeated household-based population serosurvey data. We also validated the representativeness of surveillance data from young pregnant women, aged 15 to 24 years, attending antenatal care (ANC) clinics, which UNAIDS recommends for monitoring population HIV prevalence trends in this age group. Changes in socio-demographic characteristics and reported sexual behaviour are investigated.

**Methods:**

Progress towards the UNGASS target was measured by calculating the proportional change in HIV prevalence among youth and young ANC attendees over three survey periods (round 1: 1998-2000; round 2: 2001-2003; and round 3: 2003-2005). The Z-score test was used to compare differences in trends between the two data sources. Characteristics of participants and trends in sexual risk behaviour were analyzed using Student's and two-tailed Z-score tests.

**Results:**

HIV prevalence among youth in the general population declined by 50.7% (from 12.2% to 6.0%) from round 1 to 3. Intermediary trends showed a large decline from round 1 to 2 of 60.9% (from 12.2% to 4.8%), offset by an increase from round 2 to 3 of 26.0% (from 4.8% to 6.0%). Among young ANC attendees, the proportional decline in prevalence of 43.5% (from 17.9% to 10.1%) was similar to that in the population (test for differences in trend: p value = 0.488) although ANC data significantly underestimated the population prevalence decline from round 1 to 2 (test for difference in trend: p value = 0.003) and underestimated the increase from round 2 to 3 (test for difference in trend: p value = 0.012). Reductions in risk behaviour between rounds 1 and 2 may have been responsible for general population prevalence declines.

**Conclusions:**

In Manicaland, Zimbabwe, the 2005 UNGASS target to reduce HIV prevalence by 25% was achieved. However, most prevention gains occurred before 2003. ANC surveillance trends overall were an adequate indicator of trends in the population, although lags were observed. Behaviour data and socio-demographic characteristics of participants are needed to interpret ANC trends.

## Background

In June 2001, the United Nations General Assembly Special Session (UNGASS) set a target of reducing HIV prevalence among youth, aged 15 to 24 years, by 25% in the worst-affected countries by 2005, and by 25% globally by 2010 [1]. Recently infected youth experience low HIV-related mortality [2,3]. Accordingly, changes in prevalence over time among young people should signal underlying changes in incidence. Changes in incidence are useful when gauging the effectiveness of prevention and treatment efforts [4,5].

In countries worst affected by HIV, monitoring HIV prevalence trends in the general population is a challenge. Repeated national population surveys, which can be used to construct trends in prevalence or to derive changes in estimates of age-specific incidence over time, are often too costly and complex to conduct frequently [6]. Laboratory assays to detect recent infections have so far proven unreliable in sub-Saharan Africa [7,8]. As a result, the Joint United Nations Programme on HIV/AIDS (UNAIDS) recommends using data from surveillance among pregnant women, also aged 15 to 24 years, attending antenatal care (ANC) clinics, to monitor progress toward the UNGASS target [9]. ANC surveillance data, which are available on an annual or biannual basis in most sub-Saharan Africa countries, have been found to be reasonably representative of general population prevalence, although they typically overestimate the number of infections in young people due to the selection of young women at higher risk of pregnancy and HIV infection [10-14].

Implicit in the UNAIDS recommendation is an assumption that ANC prevalence trends will mirror those among male and female youth in the general population. However, changes in sexual behaviour could cause ANC estimates to misrepresent general population trends. For example, prevention interventions promoting delays in initiating sex and/or consistent condom use could lead to general population HIV prevalence declines from reduced risk behaviour, even as prevalence at ANC clinics remains steady since, by definition, attendees are having unprotected sex. Conversely, if interventions, such as consistent condom use following HIV testing, successfully target infected individuals, a sudden drop in the ANC estimate of HIV prevalence could be observed (due to a fall in pregnancy rates in HIV-positive women) that would not be representative of the general population.

Beyond these sources of bias, ANC surveillance data are also subject to other biases that could change with time, including: clinics being sampled for convenience and that may change with time; ANC attendance varying with regard to availability and uptake; and HIV-infected women having different levels of contraceptive use and lower fertility rates [10,14-19]. To address these potential biases in ANC data, UNAIDS recommends excluding new clinics from analyses of trends, using population survey data to validate ANC estimates wherever possible, and analyzing sexual behaviour data and characteristics of the testing populations to provide context to observed changes in prevalence [6,9].

In this paper, we make use of an open-cohort, population-based household survey in Manicaland, Zimbabwe, conducted at three time intervals from 1998 to 2005 to assess directly whether the UNGASS indicator for prevalence reductions of 25% by 2005 was met among youth aged 15 to 24 years. As a secondary analysis, we also determine to what extent HIV prevalence trends in the general population mirrored those among ANC attendees, as many countries, including Zimbabwe as a whole, will not have access to repeated population survey data spanning the period covered by the UNGASS target. To validate the ANC surveillance data, we compare the proportional changes in HIV prevalence over the three rounds among pregnant women attending ANC clinics with those from the three parallel rounds of the general population survey in the same geographic areas.

Finally, we explore changes in participation, HIV prevalence by socio-demographic characteristics, such as educational status, and trends in sexual behaviour that could explain differences in the patterns of HIV estimates observed between the two datasets over time. Previous assessments in this population have shown substantial declines in population and ANC-derived HIV prevalence estimates for men and women aged 15 to 49 years in this mature epidemic, primarily linked to behaviour change [20,21].

## Methods

### Study population and data collection procedures

Data for the open-cohort, household-based population survey were collected in 12 communities in Manicaland Province, representing four geographic strata (two small rural towns, two roadside trading centres, four tea, coffee and forestry estates, and four subsistence farming areas). For the ANC surveillance, clinics offering services to pregnant women in the population survey catchment areas were selected.

Prior to each population survey round, all households and their residents were enumerated by local census. At round 1 and round 2, all men aged 17 to 54 years and women aged 15 to 44 years resident in the study households were considered eligible, except that only one member of each cohabitating or marital union was selected (at random) as eligible and, in round 2, new in-migrants were only included in communities 5 to 12. At round 3, eligibility was expanded to ages 15 to 54 years for both sexes, regardless of marital status.

In summary, the population cohort was open in nature, eligibility criteria changed over time, and individual participation could span rounds. In the parallel ANC surveillance, all women seeking ANC at participating clinics (29 in all three rounds and seven in one or two rounds only) during the population survey period (usually six to eight weeks per community) were considered eligible. Study enrolment was conditional on participants' written consent at each round, although ANC data were anonymous. The Medical Research Council of Zimbabwe and St Mary's Local Research Ethics Committee, London, provided ethical approval. Round 1 was completed from July 1998 to February 2000; round 2 began in July 2001; and round 3 began in July 2003. Further details on study methods have been published previously [20].

### HIV diagnostics

The Biomedical Research and Training Institute laboratory in Harare, Zimbabwe, performed all HIV testing. At round 1, a highly sensitive and specific (both 99.6%) dipstick-dot ICL-HIV1 & 2 Dipstick EIA was used to detect HIV antibodies [20]. Combaids-HIV-1 & 2 Dipstick was used in subsequent rounds. Apart from the principal investigators, research staff were blinded to participants' HIV status.

### Data analysis

#### Inclusion criteria

When identifying youth in the general population for inclusion in the analyses, we used two approaches. In the first, we transformed the open cohort into three cross-sectional population samples, which included all individuals aged 15 to 24 years participating in a single round only, plus one observation selected at random from those participating in multiple rounds (referred to as the "sample dataset"). This approach eliminated repeated test results for the same individual, thereby meeting the requirement of data independence for statistical testing. The total number of observations in the sample dataset was 3505 in round 1, 2151 in round 2 and 6374 in round 3.

A drawback to the sampling approach is that it could introduce a selection bias if HIV serostatus is differentially associated with the number of rounds in which an individual participates. Therefore, in a second approach, we included all men and women aged 15 to 24 years at each round, regardless of their participation in any other round (referred to as the "complete dataset"). The total number of observations in the complete dataset was 4226 in round 1, 3269 in round 2 and 7070 in round 3. While this approach captured true population point prevalence, it violated the assumption of data independence since approximately one-third of the total records belonged to individuals participating in two or more rounds. The impact of these different approaches on the study findings are considered further in the discussion.

In the ANC survey, all data from women aged 15 to 24 years seen at the 22 ANC clinics participating in all three surveillance rounds were included (i.e., data from seven clinics participating in one or two rounds were not used as recommended by UNAIDS and the World Health Organization to construct trends) [6]. The data were considered independent because very few women (5.8% in round 2 and 3.8% in round 3) reported participating in a previous surveillance round. The total numbers of participants were 671 in round 1, 624 in round 2 and 592 in round 3.

#### Statistical analyses

To describe HIV prevalence trends by data source, we calculated round-specific HIV prevalence with 95% binomial confidence intervals (CIs). CIs for round 1 and round 3 ANC estimates were adjusted for over-dispersion, as observed variance around the clinic-level estimates in these rounds was higher than expected under binomial assumptions [22]. To determine the relative proportional change in prevalence across rounds (round 1 to round 3) and between rounds (round 1 to round 2; round 2 to round 3), the difference between the earlier and the later round estimates was divided by the earlier estimate.

Confidence intervals for proportional changes using ANC data also were adjusted for over-dispersion. General population survey trends were assumed to be the "gold standard" or best representation of true underlying population prevalence in the study area; hence, the representativeness of ANC data was considered relative to that of the general population survey. Due to the rolling nature of the survey start date, the UNGASS indicator baseline measurement against which proportional prevalence change by 2005 was measured was assumed to be round 1, which spanned the period from 1998 to 2000.

When comparing proportional differences in HIV prevalence across (round 1 to round 3) and between rounds (round 1 to round 2; round 2 to round 3), we used the Z-score test-statistic. To approximate variance in these proportional differences, which was too complex to obtain analytically, we used the delta method based on the Taylor series expansion of the variance [22] The null hypothesis for trend similarity was rejected where |Z| >1.96 (i.e., p value <0.05). We adopted these approaches rather than an odds ratio to permit comparison of proportional change in HIV prevalence.

To explore whether changes in HIV prevalence within specific socio-demographic groups (such as age, marital status, education or geographic location) might be contributing to differences in intermediary trends between the sample and ANC surveillance datasets separate to or associated with changes in sexual behaviour, we similarly used a Z-score test. As an example, differences in the proportional change in prevalence trends between the two data sources (i.e., sample general population survey compared with ANC surveillance) were compared for those aged 15 to 19 years versus those aged 20 to 24 years, with the null hypothesis of no difference similarly rejected where |Z| >1.96 (i.e., p value <0.05).

Changes in behaviour between rounds in the sample dataset, including the proportion of non-sexually active youth, new partnership formation in the past year, consistent condom use among unmarried persons and partner's age for individuals reporting sex in the past two weeks were compared using a two-tailed Z-score and Student's t-tests. Behavioural data were collected using informal confidential voting interviews, which have been associated with less reported "social desirability bias" than conventional face-to-face interviewing methods in the study population [23].

The first three behavioural indicators from the survey data most closely approximate UNAIDS recommendations for monitoring behaviour change among youth as part of the 2001 UNGASS targets [9]. The fourth indicator, partner age, has been shown previously to be an important factor in HIV transmission in this population [24]. Other key factors, such as changes in sexually transmitted infections (STIs), were not investigated: biomarkers for STIs were not included in the survey, self-reported STI symptoms can be unreliable, and prevalence of STIs are thought to be low in this population [25].

## Results

### Study participants

**Figure 1:**
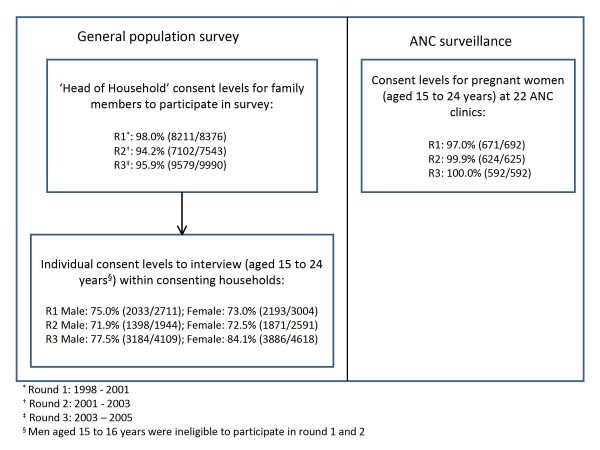
**General population survey and ANC surveillance consent levels by round in Manicaland, Zimbabwe, 1998-2005**.

Figure 1 shows the results of household- and individual-level consent in the population survey and ANC surveillance datasets by round.

Enrolment in the population survey was high, with more than 94% of households agreeing to participate in each round. Among youth in the participating households, consent levels were similar for males and females, except that fewer males (77.5%) than females (84.1%) participated in round 3 (p value <0.001). In the ANC surveillance, participation was nearly universal (97.0%-100%). The population survey distribution reflected the number of study sites, with 36.3% of participants living in subsistence farming areas, 28.9% in estates, 19.8% in roadside trading centres, and 15.9% in towns aggregated across all rounds. In the ANC survey, 31.8% of participants attended clinics in subsistence farming areas, 34.4% in estates, 14.8% in roadside trading centres, and 19.0% in towns.

Across rounds, the mean age of individuals in the population survey sample dataset was younger (19.2 years) than that in the ANC survey (20.2 years) (p <0.001). Similar mean ages were recorded in round 2 and round 3; in the latter, the eligibility criteria were expanded to include men aged 15 to 16 years. Reflecting their younger ages and the inclusion of men, fewer individuals in the population survey were married (13.2% versus 75.6% in ANC surveillance, p <0.001), but more had secondary or higher education (81.7% versus 63.7% in ANC surveillance, p <0.001).

The sex ratio (males/females) fluctuated over time in the population survey sample dataset from 0.95 in round 1 to 0.76 in round 2 and 0.83 in round 3. ANC attendance among women in the population survey sample who were currently pregnant or had completed a pregnancy in the six months before the survey date was 80.6% in round 1, 81.3% in round 2 and 85.0% in round 3. Of those seeking antenatal care, approximately 80% at each round attended their local clinic. Overall, 13.0% of sexually active women in round 1, 9.2% in round 2 and 18.9% in round 3 reported a recent or current pregnancy. Similar distributions were observed in the complete population dataset.

### Population-based and ANC HIV prevalence among youth

**Figure 2:**
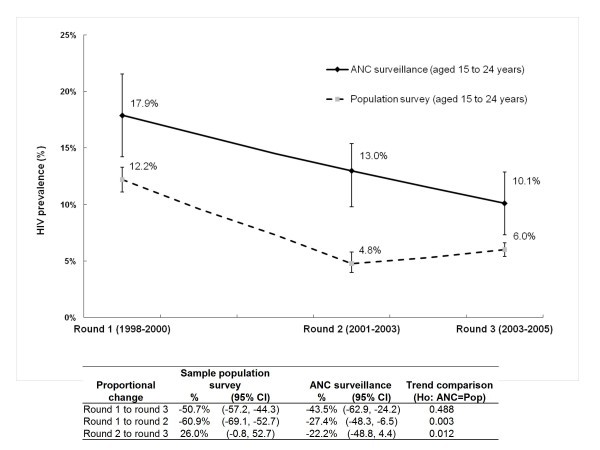
**HIV prevalence among young men and women aged 15-24 years in the sample population survey dataset and from ANC surveillance from 1998 to 2005, Manicaland, Zimbabwe**.

**Figure 3:**
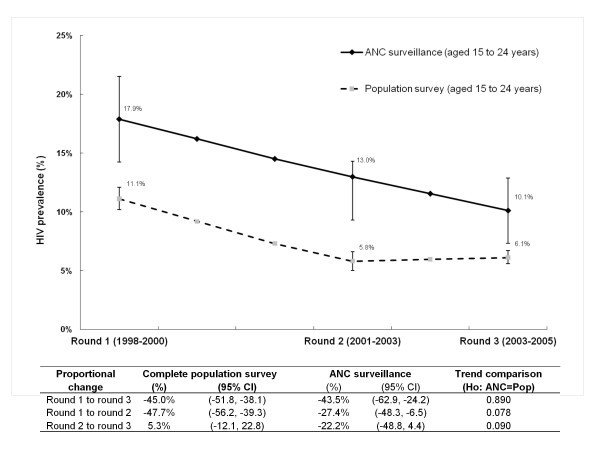
**HIV prevalence among young men and women aged 15-24 years in the complete population survey dataset and from ANC surveillance from 1998 to 2005, Manicaland, Zimbabwe**.

Figures 2 and 3 summarize HIV prevalence levels and trends among youth in the general population survey from 1998 to 2005 in the sample and complete datasets respectively. Levels and trends from the ANC surveillance for the same time periods are also shown.

In general, population prevalence was lower than ANC prevalence at each round, reflecting the increased risk of HIV infection in young women as compared with young men in this population, and the selection for high-risk sexual activity that exposes women to both pregnancy and HIV infection.

With regard to the UNGASS indicator, proportional HIV prevalence (as summarized in the table accompanying Figure 2a) declined by 50.7% (95% CI: -57.2%, -44.3%) in the sample general population dataset, from 12.2% in round 1 to 6.0% in round 3. This was similar to the reduction of 43.5% (95% CI: -62.9%, -24.2%) in the ANC surveillance, from 17.9% in round 1 to 10.1% in round 3 (test for difference in trend, p value = 0.488) (see Figure 2a). Reductions from both data sources exceeded the UNGASS target of 25% by 2005. Despite the overall similarities, there were differences in intermediary HIV prevalence trends. From round 1 to round 2, the proportional reduction in the ANC data of 27.4% (from 17.9% to 13.0%) was half that in the general population of 60.9% (from 12.2% to 4.8%) (test for difference in trend, p value = 0.003). From round 2 to round 3, HIV prevalence declined further in the ANC data, by 22.2% (from 13.0% to 10.1%), but rose in the general population, by 26% (from 4.8% to 6.0%) (p value = 0.012).

Similar round 1 to round 3 declines of 45.0% (95% CIs: -51.8%, -38.1%) were also observed in the complete population data set as compared with the 43.5% reduction (95% CI: -62.9%, -24.2%) in the ANC surveillance data (test for difference in trend, p value = 0.890) (see the table accompanying Figure 2b). Unlike the sample data set, however, intermediary differences were not statistically significant (round 1 to round 2, p = 0.078; round 2 to round 3, p = 0.090). Nevertheless, HIV prevalence rose minimally by 5.3% from round 2 to round 3 in the population data at a time when the ANC estimates declined by a further 22.2%, providing some evidence, albeit non-significant, for differences in ANC and general population prevalence trends for the complete data set, as well.

### Socio-demographic predictors of trend differences

Using the Z-score test statistic to compare proportional changes in intermediary HIV prevalence trends (e.g., round 1 to round 2 and round 2 to round 3) from the ANC surveillance and sample population dataset by socio-demographic strata (e.g., 15-19 year olds versus 20-24 year olds), we observed the patterns of change to be broadly similar (p values for differences in the proportional changes in intermediary prevalence trends between socio-demographic groups >0.10, except for education status where p value = 0.092) (see Table 1). This finding suggests that the significant round-to-round changes in the non-stratified trends did not occur within one particular socio-demographic group, but rather across the population as a whole.

**Table 1:** HIV prevalence estimates by socio-demographic characteristics among youth (aged 15-24 years) in the sample general population survey and ANC surveillance in Manicaland, Zimbabwe, 1998-2005*

	Sample general population survey			ANC surveillance		
	(Aged 15-24 years)			(Aged 15-24 years)		
HIV prevalence estimates by socio-demographic characteristics	Round 1 (1998-2000) HIV % (n/N)	Round 2 (2001-2003) HIV % (n/N)	Round 3 (2003-2005) HIV % (n/N)	Round 1 (1998-2000) HIV % (n/N)	Round 2 (2001-2003) HIV % (n/N)	Round 3 (2003-2005) HIV % (n/N)
**Age**						
15-19 years	4.5 (72/1600)	1.9 (25/1328)	2.6 (96/3738)	12.6 (33/261)	7.8 (19/245)	4.6 (11/242)
20-24 years	18.6 (354/1905)	9.6 (79/823)	10.9 (288/2636)	21.2 (87/410)	16.4 (62/379)	14.0 (49/350)
**Gender**						
Male^†^	6.2 (106/1712)	2.3 (21/929)	2.7 (77/2886)			
Female	17.9 (320/1793)	6.8 (83/1222)	8.8 (307/3488)	17.9 (120/671)	13.0 (81/624)	10.1 (60/592)
**Education**						
None/primary	18.9 (155/819)	11.3 (37/329)	10.8 (113/1043)	18.3 (49/268)	11.3 (24/213)	10.8 (22/204)
Secondary/higher	10.1 (271/2686)	3.6 (66/1819)	5.0 (263/5287)	17.7 (71/402)	13.9 (57/411)	9.8 (38/388)
**Residence**^‡^						
Town	18.5 (109/588)	10.1 (31/308)	9.7 (99/1016)	19.7 (23/117)	12.3 (14/114)	9.4 (12/128)
Commercial estate	12.2 (140/1152)	5.3 (33/618)	7.0 (111/1595)	19.0 (41/216)	14.6 (32/220)	11.3 (24/213)
Subsistence farm	8.8 (103/1174)	3.5 (28/806)	4.4 (105/2386)	18.4 (40/217)	11.8 (26/220)	11.7 (19/163)
Roadside trading	12.5 (74/591)	2.9 (12/419)	5.0 (69/1377)	13.2 (16/121)	12.9 (9/70)	5.7 (5/88)

* P value results of Z-score tests for differences in the proportional change in prevalence trends between the two data sources (i.e., sample general population survey compared to ANC surveillance) by socio-demographic groupings (e.g., those aged 15-19 years versus those aged 20-24 years) for round 1 to round 2 and round 2 to round 3 were highly non-significant (p value >0.10), except for HIV prevalence trends by educational status where p = 0.092. As there was no evidence for any differences in trends by socio-demographic groupings, these results are not presented.

^†^Men aged 15-16 years were ineligible to participate in rounds 1 and 2.

^‡ ^In ANC surveillance, "Residence" indicates the location of the ANC clinic where the woman sought prenatal services and not necessarily where she resides.

### Behavioural risk factors

Table 2 shows trends in selected behavioural indicators reported in the general population sample dataset that could explain observed intermediary differences.

**Table 2:** Selected behavioural indicators among youth (aged 15-24 years) in the sample general population survey in Manicaland, Zimbabwe, 1998-2005

	Round 1 (1998-2000)		Round 2 (2001-2003)		Round 3 (2003-2005)		Round 1 to round 2 p values	Round 2 to round 3 p values
**Individuals not yet initiating sex (%, n/N)**								
Male								
15-19 years of age*	49.4	(348/704)	69.5	(348/501)	76.3	(1475/1931)	**<0.001**	**0.002**
20-24 years of age	13.5	(136/1008)	18.2	(78/428)	22.2	(240/1079)	**0.021**	0.085
Female								
15-19 years of age	66.0	(591/896)	79.4	(657/827)	76.4	(1475/1931)	**<0.001**	0.079
20-24 years of age	9.5	(85/897)	19.2	(76/395)	11.8	(183/1557)	**<0.001**	**<0.001**
**Number of new partners among those having sex in the last year (%, n/N)**								
Male								
0	27.9	(276/989)	42.1	(150/356)	32.6	(309/947)	**<0.001**	**0.001**
1	39.4	(395/989)	37.4	(133/356)	47.2	(447/947)	0.507	**0.002**
2+	32.2	(318/989)	20.5	(73/356)	20.2	(191/947)	**<0.001**	0.905
Female								
0	69.2	(639/923)	74.0	(304/411)	76.4	(1224/1602)	0.075	0.310
1	27.2	(251/923)	23.8	(98/411)	21.7	(348/1602)	0.192	0.360
2+	3.6	(33/923)	2.2	(9/411)	1.9	(30/1602)	0.379	0.696
**Partner's mean age and 95% CIs (in years) for those reporting sexual intercourse in the past two weeks**								
Male	18.9	(18.5-19.2)	19.0	(18.5-19.4)	19.4	(19.1-19.8)	0.773	0.158
Female	28.8	(28.1-29.4)	27.4	(26.7-28.1)	27.6	(27.3-28.0)	**0.010**	0.552
**Consistent condom use with the last partner in the previous two weeks among unmarried individuals**								
Male	60.9	(145/238)	63.0	(46/73)	65.1	(123/189)	0.748	0.754
Female	36.8	(28/76)	45.2	(14/31)	45.5	(30/66)	0.424	0.978

*Men aged 15-16 years were ineligible to participate in rounds 1 and 2

For both sexes and age groups, the proportion of those not yet initiating sex increased significantly from round 1 to round 2. From round 2 to round 3, a smaller but still significant increase was observed among younger men (69.5% to 76.3%; p value 0.002), although this likely reflected the inclusion of men aged 15 to 16 years old in the survey at round 3. For women, a large reduction of those not yet initiating sex was seen among older women (19.2% to 11.8%; p value <0.001). For those who had sex within the past year, the number of men reporting no new partners increased from round 1 to round 2 (27.9% to 42.1%; p value <0.001), but declined from round 2 to round 3 (42.1 to 32.6%; p value = 0.001).

Women experienced a steady increase in the proportion reporting no new partners (from 69.2% in round 1 to 76.4% in round 3); however, round-to-round increases were not significant. Estimates of mean partner age of persons having sex in the past two weeks and consistent condom use among unmarried men generally tended toward less risky behaviour; however, only the reduction in mean partner age among women from 28.8 years in round 1 to 27.4 years in round 2 was statistically significant (p value = 0.010).

## Discussion

Our results show that the UNGASS target of a reduction of 25% in HIV prevalence by 2005 among young men and women aged 15 to 24 years was achieved in Manicaland, Zimbabwe, with reductions by 2005 nearly twice the targeted value. For both the sample and complete population-based datasets, the lower bounds of the 95% confidence intervals for round 1 to round 3 proportional reductions comfortably exceeded 25%. Despite this achievement, from the analysis of intermediary trends, it is evident that these declines have not been consistent over time. Reductions were greatest prior to 2003, most likely reflecting the rapid expansion and impact of HIV prevention campaigns in the early 2000s throughout the country [26,27]. As was the case in Uganda, another sub-Saharan Africa country with high prevalence early on in the epidemic, a visible increase in HIV-related mortality in the late 1990s among the participating communities also may have accelerated early behaviour change among youth [26].

Subsequent to 2003, however, the increase in prevalence could indicate that prevention efforts may have been less effective in reaching high-risk youth. This rise was accompanied by significant increases in the number of women aged 20 to 24 years initiating sex and an increase in the number of sexually active men with one new partner in the past year, and it took place despite the inclusion of young men aged 15 to 16 years in round 3 who are typically at lower risk of HIV infection compared with their female counterparts and men aged 17 years and older.

While our results suggest that behaviour change has been the driving force behind the observed trends, it is also possible that these changes could reflect shifts in the direction and magnitude of bias in the data. We assume that population survey estimates are representative of underlying population prevalence in the study area and that any biases in these estimates are stable with time. With regard to this assumption, however, two possible concerns could be raised.

First, participation levels and eligibility criteria changed across rounds of the general population survey, and these changes could have distorted our representation of true underlying population prevalence in the study area. Acceptance levels, however, are consistent with those achieved in other HIV population surveys [28], which have been shown to produce minimally biased HIV prevalence estimates [29]. Land reform and migration, coinciding with round 2, could have also caused variation in the composition of (particularly male) participants across rounds and skewed HIV prevalence estimates in this round in particular. However, individuals migrating to more urban areas during this period did not have higher levels of HIV prevalence [30].

In addition, the inclusion of men aged 15 to 16 years caused a significant increase in the percent of men aged 15 to 19 years not yet initiating sex; nevertheless, exclusion of these men from the analysis did not change the overall conclusions. Given these findings, we are reasonably confident that HIV prevalence trends among youth reflected those of the underlying population study area. However, additional survey data from two upcoming rounds (round 4: 2006-2008; and round 5: 2009-2011) will provide for a stronger indication of overall trends, as well as the opportunity to directly measure changes in incidence.

Second, the two methods we used for constructing the general population data sets when analyzing trends also could have distorted our estimates. For example, the sampling approach using the three independent data sets led to a slight overstatement of population HIV prevalence in round 1 (risk ratio, RR, of sample prevalence divided by complete prevalence = 1.10), a more pronounced understatement of population prevalence at round 2 (RR = 0.83) and minimal bias in round 3 (RR = 0.98) since participation in multiple rounds was correlated with HIV status. Accounting for this bias, our sample estimates would have exaggerated the proportional decline from round 1 to round 2 by 27% and overstated the increase from round 2 to round 3 by 15%. In the second approach, repeated testing on the same individuals across rounds would have overstated the precision associated with the trends. Additional research is needed to improve the statistical analysis of trends measured in cohort surveys since none of the approaches explored were without limitation.

As most countries will not have access to repeated population survey data, the results of our secondary analysis, showing that ANC-based surveillance data broadly reflected the overall change in HIV prevalence among young men and women in the general population between 1998 and 2005, are encouraging. Despite this, the ANC estimates did fail to capture short-term or intermediary changes occurring in the general population, especially in the sample data set. The ANC data indicated a consistent steady decline in HIV prevalence from round 1 to round 2 to round 3, while a rapid fall was observed in the general population between round 1 and round 2, followed by a slight increase through round 3.

The intermediary divergence in trends is important to explore in this population because policymakers, who have typically relied on ANC surveillance data to measure the impact of interventions in Zimbabwe, could have underestimated the effectiveness of early HIV prevention programmes that were scaled up in the late 1990s [31], but then overestimated their subsequent impact at a time when resources could have been used elsewhere or in more effective ways. Notably, the slow, steady decline in ANC prevalence observed here resembles that seen in national ANC surveillance data from 2000 to 2006 among those aged 15 to 24 years [32], suggesting that national-level estimates of trends in HIV incidence among youth could have been similarly distorted and incorrect conclusions drawn about the effectiveness of prevention interventions. A similar study from Lusaka, Zambia, also comparing trends in the general population and among ANC attendees found that HIV prevalence among youth between 1995 and 2003 declined more rapidly than among ANC attendees due to increases in educational attainment leading to postponement in ages at first sex and first pregnancy [33].

As was the case in Lusaka, the most reasonable explanation for these divergences is the previously described changes in sexual behaviour in the general population that would not have been reflected among ANC attendees. Primarily, the postponement of sexual debut and, to a lesser extent, reductions in the number of new partners and the age of partners, and increases in consistent condom use among youth generally from round 1 to round 2 could have rapidly reduced HIV transmission in this population while having a more limited impact on the declining fraction who continued to become pregnant by practicing unprotected sex.

Mathematical modelling by Zaba and colleagues supports this hypothesis, showing how young pregnant women become increasingly less representative of the general population with regard to their sexual behaviour as the age of sexual debut increases and risk of HIV transmission declines [12]. The more gradual reductions in HIV prevalence seen in the ANC data, which contrast with Zaba and colleagues' results, may reflect the benefits to young pregnant women of the reduced circulation of HIV in the adult population that occurred from round 1 to round 2 [20].

Other factors that could have contributed to the contrasting temporal patterns of change in HIV prevalence seen in the general population and ANC data include changes in the profile of women accessing ANC services. However, we observed only minor increases in ANC uptake from 80.6% (round 1) to 81.3% (round 3) and the proportion attending their local ANC remained steady at around 80%. Very few pregnant women refused to participate in the ANC surveys. Scale up of HIV testing and prophylaxis services for pregnant women could result in a selective increase in uptake of ANC services by HIV-positive women; however, in Mwanza, Tanzania, while the quality and type of ANC services influenced where women sought prenatal care, these preferences were not differentially associated with a woman's HIV status [34].

Furthermore, our study occurred during a period when HIV testing and prophylaxis services for pregnant women in Zimbabwe were limited; thus, a selective increase in uptake of ANC services by HIV-positive women is unlikely. Examination of access to ANC services and the characteristics of women seeking these services over time are nonetheless recommended as these may shift with time, particularly if HIV prevention and treatment programmes become more closely integrated with family planning efforts [35]. Finally, as antiretroviral therapy and, by extension, the number of years a person lives with HIV increases, prevalence trends may become a less accurate indicator of underlying incidence, especially if more recently infected individuals are placed on treatment. Methods for adjusting prevalence trends to reflect changes in survivorship bias over time will be needed.

## Conclusions

In conclusion, this analysis of data from Manicaland, Zimbabwe, shows several important findings. First, for a population that has been greatly affected by HIV, substantial and successful efforts toward preventing new infections among youth aged 15 to 24 years were made in the late 1990s and early 2000s. The effects of prevention efforts in the general population appear to have stalled somewhat after 2003, although declines among young women attending ANC clinics were still evident and the UNGASS target for 2005 was reached.

Second, trends in reported sexual behaviour, rather than biases in the population survey data, seem the most likely explanation for these declines. As a result, trends in prevalence likely reflect trends in underlying population prevalence and incidence.

Finally, although, in general, the evidence for the usefulness of ANC surveillance data to monitor HIV prevalence trends among youth in this eastern Zimbabwe population is encouraging, intermediary trends were found to differ. Behavioural data collected in the population survey were critical to interpreting these differences, however, so caution should be exercised when interpreting ANC trends without broader indicators of population-level behaviour risk. In addition, we highlight the possible role that increased access to integrated pre-natal HIV prevention and treatment interventions could play in changing the profile of women seeking ANC services over time, thereby possibly exacerbating differences in prospective trends. Examination of access to ANC services and the characteristics of women seeking these services over time merits more careful consideration in future studies.

## Competing interests

The authors declare that they have no competing interests.

## Authors' contributions

KAM, with significant input from CAN, CAD, JMGC and SG, originally conceived of and designed the analysis and drafted the article. CAN, EM, PM and SG contributed to the collection and assembly of the data. All authors actively participated in the analysis and interpretation of the data and critical revision of the draft article. All authors approved the final submission of the article and its contents.
